# Complete Genome Sequence of *w*Ana, the Wolbachia Endosymbiont of Drosophila ananassae

**DOI:** 10.1128/MRA.01136-19

**Published:** 2019-10-24

**Authors:** Mark T. Gasser, Matthew Chung, Robin E. Bromley, Suvarna Nadendla, Julie C. Dunning Hotopp

**Affiliations:** a Institute for Genome Sciences, University of Maryland School of Medicine, Baltimore, Maryland, USA; b Department of Microbiology and Immunology, University of Maryland School of Medicine, Baltimore, Maryland, USA; c Greenebaum Cancer Center, University of Maryland, Baltimore, Maryland, USA; Broad Institute

## Abstract

Here, we present the complete genome sequence of the Wolbachia endosymbiont *w*Ana, isolated from Drosophila ananassae and derived from Oxford Nanopore and Illumina sequencing. We anticipate that this will aid in *Wolbachia* comparative genomics and the assembly of *D. ananassae* specifically in regions containing extensive lateral gene transfer events.

## ANNOUNCEMENT

Lateral gene transfer (LGT) from the Wolbachia endosymbiont *w*Ana in Drosophila ananassae constitutes >2% of the insect genome, including integrations of multiple *w*Ana genomes in the abnormally large, largely heterochromatic chromosome 4 (1, 2). To aid in studies of this massive LGT, the complete *w*Ana genome was obtained.

**FIG 1:**
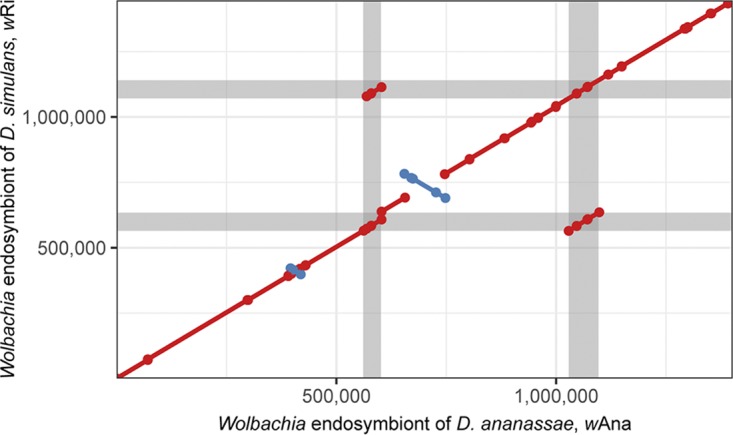
Synteny between *w*Ana and *w*Ri. A MUMmer plot between the genomes of *w*Ana and the *Wolbachia* endosymbiont of *D. simulans*, *w*Ri, was generated using NUCmer to assess synteny. Red and blue line segments are indicative of conserved regions between the two strains, with blue lines being inverted in *w*Ri relative to *w*Ana. The gray-shaded regions mark the two duplicate regions in the *w*Ana genome and *w*Ri genomes where there is a deletion in *w*Ana relative to *w*Ri.

To generate an LGT-free line of *D. ananassae*, Michael Clark and John Werren at the University of Rochester introgressed *D. ananassae* harboring the *w*Ana Hawaii strain into the LGT-free *D. ananassae* Florida line for 10 generations to create *D. ananassae* W2.1, which was obtained from Irene Newton at Indiana University in Bloomington. The line was reared on molasses medium in plugged bottles at 25°C and 70% relativity humidity with a 12/12-h light/dark cycle. The whole flies were flash frozen in liquid nitrogen in a 50-ml Falcon tube and vortexed for 3 s, and the headless bodies were then collected with a small brush. High-molecular-weight DNA was isolated from the adult Drosophila ananassae W2.1 bodies using phenol-chloroform extraction with ethanol precipitation with sodium acetate (3). Illumina paired-end (2 × 150-bp) library construction and sequencing was performed using the Nextera XT library prep protocol on an Illumina MiSeq platform, yielding 51.4 Gbp in 340,594,990 sequenced reads. Long-read library preparation (SQK-RAD004) and sequencing (FLO-MIN106 R9 MinION) protocols from Oxford Nanopore Technologies (ONT) were performed with slight modifications using 2 μg of DNA as the input and omitting library-loading beads. Raw ONT read signals were base called using Albacore v2.3.1, which yielded 864.5 Mbp in 87,410 reads without barcoding or multiplexing. Sequencing adapters and possible chimeras were removed from base-called reads with Porechop v0.2.3 (4) using –discard_middle. An initial *de novo* assembly using only the ONT reads and miniasm v0.2 (5) yielded *Drosophila* and *Wolbachia* contigs. From the assembly, a single contig of the complete *w*Ana genome was identified by aligning to the *w*Ri genome (6) using MUMmer v3.0 (7). Illumina and ONT reads mapping to this putative *Wolbachia* contig were identified using BWA aln/sampe (8) and Minimap2 v2.10 (9) with -ax map-ont, respectively. The *Wolbachia*-mapping reads were used to construct a new, hybrid *de novo* assembly using Unicycler v0.4.4 (10). The assembly was visually inspected for misassemblies by remapping Illumina and ONT reads to the hybrid *de novo* assembly. The *w*Ri genome has two nearly identical 68-kbp regions that both include a prophage (Fig. 1). We identified a 28-kbp deletion at the end of the first of these duplicated regions in the *w*Ana genome (Fig. 1). This deletion was supported by ONT reads that spanned the deleted region but failed to assemble correctly. Therefore, the correct sequence of the first duplicate region was manually inserted after being derived from the spanning ONT reads that were Illumina corrected with Pilon v1.22 (11) and manually inspected for errors. The final, complete, and corrected assembly of the *Wolbachia* endosymbiont of Drosophila ananassae, *w*Ana, consists of a circular chromosome of 1,401,460 bp (GC content, 35.2%) with average sequencing depths of ∼1,240× and 12× for the Illumina and ONT reads, respectively. The genome was annotated using the IGS Prokaryotic Annotation Pipeline (12) with Prodigal v2.6.3 set to disallow calling genes that run off the edge of contigs (13). All software was run using default settings unless otherwise noted. The *w*Ana genome contains 1,289 open reading frames (ORFs), 35 tRNA genes, and one copy of each of the 5S, 16S, and 23S rRNA genes.

### Data availability.

The complete genome sequence of *w*Ana has been deposited in GenBank under the accession number CP042904. The Oxford Nanopore FASTQ file, Oxford Nanopore FAST5 file, and Illumina sequencing reads are available from the NCBI Sequence Read Archive (SRA) under the accession numbers SRR8306005, SRR9866440, and SRR8278850, respectively.
